# Effect of Annular Laser Metal Deposition (ALMD) Process Parameters on Track Geometry and Thermal History on Ti6Al4V Alloy Clad

**DOI:** 10.3390/ma16114062

**Published:** 2023-05-30

**Authors:** Jinchao Zhang, Yupeng Cao, Heng Wang, Tuo Shi, Boyong Su, Lei Zhang

**Affiliations:** 1 School of Mechanical Engineering, Nantong University, Nantong 226019, China; zhangjc@ntu.edu.cn (J.Z.); wanghengntu@163.com (H.W.); sby1872@163.com (B.S.); zhangleint@ntu.edu.cn (L.Z.); 2 School of Optoelectronic Science and Engineering, Soochow University, Suzhou 215021, China; shituo@suda.edu.cn

**Keywords:** laser metal deposition, track geometry, thermal history, annular laser beam, Ti6Al4V

## Abstract

Annular laser metal deposition (ALMD) is a rising technology that fabricates near-net-shaped components. In this research, a single factor experiment with 18 groups was designed to study the influence of process parameters on the geometric characteristics (bead width, bead height, fusion depth, and fusion line) and thermal history of Ti6Al4V tracks. The results show that discontinuous and uneven tracks with pores or large-sized incomplete fusion defects were observed when the laser power was less than 800 W or the defocus distance was −5 mm. The laser power had a positive effect on the bead width and height, while the scanning speed had the opposite effect. The shape of the fusion line varied at different defocus distances, and the straight fusion line could be obtained with the appropriate process parameters. The scanning speed was the parameter that had the greatest effect on the molten pool lifetime and solidification time as well as the cooling rate. In addition, the microstructure and microhardness of the thin wall sample were also studied. Many clusters with various sizes in different zones were distributed within the crystal. The microhardness ranged from 330 HV to 370 HV.

## 1. Introduction

Laser metal deposition (LMD) has attracted attention due to its potential to reduce lead times, material waste, and overall manufacturing costs [1,2,3]. LMD is a process whereby parts are fabricated by the layer-by-layer depositing and joining of material with a laser as an energy source. High process flexibility makes LMD a possible solution in the large-scale production of components for the repair as well as the deposition of functionally graded materials [4,5]. Ti6Al4V, as a typical (α+β) phase titanium alloy, has been widely used in the aerospace, ship, and biomedical industries due to its high specific strength and good biocompatibility, as well as its superior corrosion resistance [6,7,8]. Hence, the development of Ti6Al4V by LMD components is of great interest, especially to the aerospace industry. The LMD-based fabrication of titanium alloys has focused on geometry, residual stresses, microstructures, and mechanical properties.

The LMD process involves various energy transfer mechanisms, which are dependent on process parameters, such as laser type, laser power, spot size, scanning speed, powder feed rate, and so on. These factors are responsible for the temperature of the molten pool and ultimately affect the track geometry in terms of its width, height, fusion depth, dilution rate, and wetting angle [9,10]. In addition, an unsuitable process parameter combination will result in bad metallurgical bonding between the track and the substrate, which is detrimental to multi-layer, multi-track manufacturing [11,12]. Therefore, the research on the effect of process parameters on the geometry and properties of the clad track is necessary. Zhao et al. [13] investigated the effect of process parameters on the geometry of the YCF104 clad track. Manjaiah et al. [14] analyzed the geometry, surface waviness, and microstructures of deposited 316L stainless steel; the results showed that the scan speed and laser power presented contrasting effects on the shape and morphology of the track, and columnar and homogeneous cellular dendritic grains were observed. Sandeep et al. [15] developed a 3D thermal model to correlate the temperature with deposition quality. Bennett et al. [16] investigated the effect of powder flow and laser power on the thermal history and dimensions of deposited IN718 tracks, and the relationship between thermal history and track height was formed. Foteinopoulos et al. [17] investigated the effect of scanning strategy on thermal stresses and deformations. Zhang et al. [18] used a predictive model to simulate thermal history and microstructure evolution, and the grain morphology result was in accordance with the experiment.

Annular laser metal deposition (ALMD) is a method in which the sprayed powder is in the middle of the annular laser beam obtained by optical path conversion; the opposite is the case in LMD, where the solid laser beam is in the middle while the sprayed powder is on the outside [19]. ALMD has several advantages, such as high nozzle-inclined angles, good surface finish, and relatively uniform energy distribution of the molten pool [20,21]. There is still insufficient information regarding the geometrical properties and thermal history of the track during the ALMD process. In this study, substantial experiments were carried out to investigate the effect of the process parameters on the track geometry and thermal history of the Ti6Al4V alloy. In addition, the microstructure and microhardness of the thin wall sample were assessed. The Ti6Al4V alloy successfully produced by ALMD can be applied to form or repair high value-added components.

## 2. Materials and Methods

### 2.1. Materials and Experimental Setup

Gas-atomized spherical Ti6Al4V powder was used as feedstock powder. The range of particle size of the powders was 75 to 106 µm. The Ti6Al4V rolled plate with a thickness of 8 mm was used as the substrate. The chemical compositions of the substrate material and Ti6Al4V powder are listed in Table 1. Before the experiments were conducted, the powders were dried in a vacuum oven at 120 °C for 2 h to remove moisture, and the substrate was cleaned with acetone to remove dirt and oil particles.

**Figure 1:**
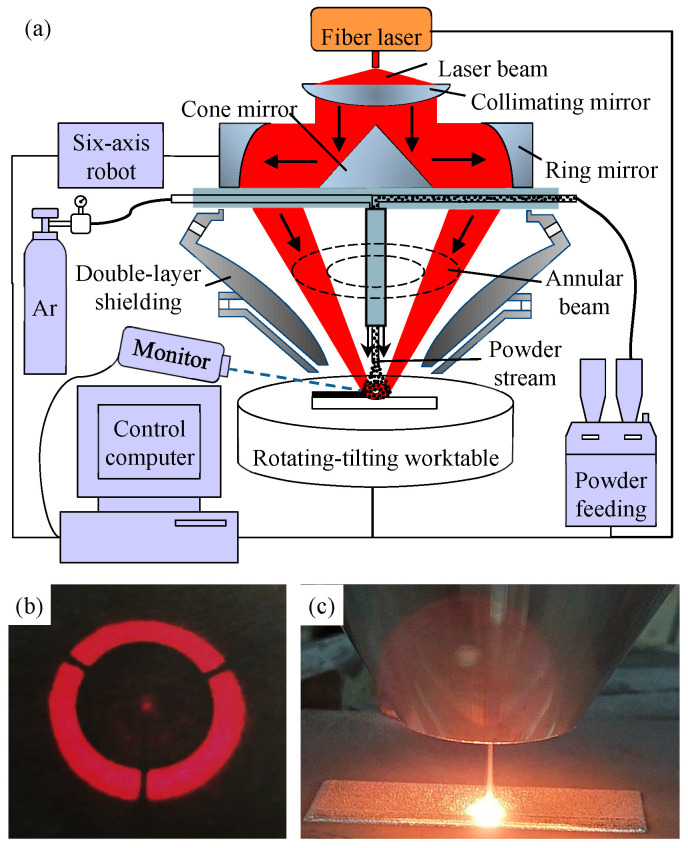
ALMD technology: (**a**) the schematic diagram of ALMD system; (**b**) the annular laser spot; (**c**) the ALMD process.

All the specimens were produced using annular laser metal deposition (ALMD), as shown in Figure 1a. The ALMD system included a 2 kW fiber laser (IPG YLS-2000-TR, IPG Photonics, Oxford, MA, USA), a six-axis mobile robot (KUKA KR 60-3F, TIE Industrial, La Vergne, TN, USA), a powder feeder (GTV PF2/2, GTV, Luckenbach, Germany), and a self-developed annular beam powder feeding nozzle [22]. The annular laser beam was obtained by an optical path conversion that was first diffused by cone mirror and then focused by ring mirror, as shown in Figure 1b. A single powder feeding tube was installed coaxially in the middle of the annular beam, and thus, the powder stream was surrounded by the annular laser, in contrast to the traditional powder feeding nozzle of LMD, in which the solid laser beam is in the middle of the oblique powder streams. In order to protect the ALMD process from atmospheric contamination in an open environment, a developed coaxial double-layer shielding device was employed [23] to generate a local inert atmosphere around the molten pool, replacing the common sealed chamber. The whole ALMD process was steady, and there was no significant spatter formation, as shown in Figure 1c.

### 2.2. Experimental Procedure and Investigation Methods

A two-stage experiment was conducted to investigate the effects of the ALMD processing parameters. In the first stage, a single clad was deposited to analyze the effect on bead geometry and quality. In the second stage, multiple layers were deposited to observe the interactions between layers.

For the first stage of the experiment, the laser power (700~1200 W), scanning speed (3~8 mm/s), and defocus distance (0~−5 mm) were selected as the dependent variables, based on a preliminary experiment. Table 2 shows the experimental design of the ALMD, which had three factors and six levels. A total of eighteen experiments were carried out, varying one parameter at a time. The laser power varied at intervals of 100 W, the scanning speed varied at intervals of 1 mm/s, and the defocus distance varied at intervals of −1 mm. The other fixed processing parameters were as follows: the powder feeding rate was 1.4 g/min, the total shielding gas flow rate was 30 L/min (the internal and external shielding gas flow rates were 18 L/min and 12 L/min, respectively), and the length of deposited track was 20 mm. Argon gas was used as a carrier and shielding gas to transfer the feedstock powders and also to protect the molten pool from oxidation.

**Figure 2:**
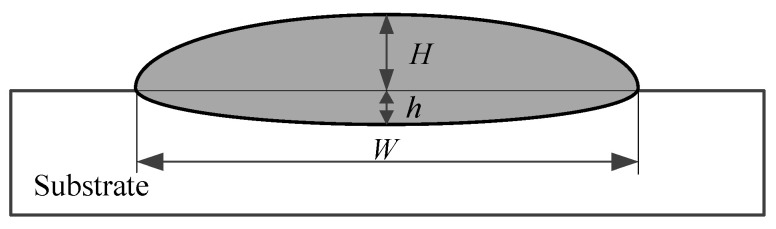
The geometrical characteristics of the specimen (*W* is bead width, *H* is bead height, and *h* is fusion depth).

The clad quality of the specimens was evaluated using multiple criteria, such as bead morphology, bead width, bead height, and fusion depth. A bead with a continuous and uniform morphology was considered beneficial to the subsequent deposition. The bead width, bead height, and fusion depth were measured to study the influence of the process parameters on the geometrical characteristics. The measurements were taken by Image J (https://github.com/imagej) and are labeled in Figure 2.

The specimens were cut in the middle of the clad tracks to observe the geometric dimensions. Prior to microscopy, the samples were prepared by a standard mechanical polishing method and etched through a Kroll reagent consisting of 1 mL HF, 2 mL HNO_3_, and 50 mL H_2_O. The optical microscope (OM) and scanning electron microscope (SEM) were used to obtain qualitative and quantitative observations, including geometrical characteristics, as well to observe the microstructure, porosity, and lack of fusion in the bead.

For the second stage of the experiment, a thin wall specimen with 15 layers was built to observe the resulting geometry, macrostructure, and microstructure. In order to ensure that the lifting height of the robot was in accordance with the deposition height of the previously built layer during the whole process, a layer height monitor device was used to dynamically adjust the robot’s movement, rather than a constant lift amount [24]. The sample was prepared in the same way as the first stage of the experiment for OM and SEM observation. In addition, the microhardness of the specimen in the vertical direction was measured by a MH-5 Vickers tester using a load of 0.5 kg for 15 s.

### 2.3. Temperature Measurement

During the ALMD process, the molten pool temperature was monitored with a non-contact two-color infrared pyrometer (Metis M322). The pyrometer was aligned to the upper surface of the substrate to measure the molten pool temperature at a fixed point. The temperature range of the pyrometer was between 800 and 3000 °C, the response time was less than 1 ms, and the highest acquisition frequency reached 3300 values/s. When the annular laser beam scanned through the middle point of the clad, the changing temperature changing was recorded by thermometric system. In this work, the thermal history of the midpoint of the track under different process parameters (in Table 2) was measured for further analysis and comparison.

## 3. Results and Discussion

### 3.1. Effect of Process Parameters on Morphology

**Figure 3:**
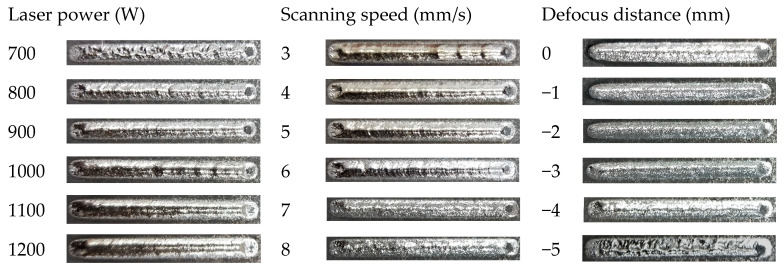
Surface morphologies of single tracks fabricated by ALMD at different process parameters.

The surface morphologies of the Ti6Al4V clads fabricated by ALMD at the different parameters are shown in Figure 3. All the laser powers, scanning speeds, and defocus distances had an important influence on the formation of the single tracks. For the low laser power, high scanning speed, and defocus distance (that is, the big spot diameter), discontinuous and uneven tracks were observed. Due to insufficient energy input, the lifetime of the molten pool is short, which leads to a lack of bonding between the track and the substrate, insufficiently melted powders, and weak melt convection [25,26,27]. When the energy input is sufficient, the track is smooth and continuous. The surface of the track presented metallic silver, and no obvious macroscopic forming defects could be found.

It can also be seen from Figure 3 that the surface of the track appears light yellow, indicating a slight oxidation, when the laser power exceeds 1100 W, or the scanning speed is 3 mm/s. In addition, when the scanning speed exceeds 7 mm/s, the surface of the track is rough, and a large number of non-melted or partially melted powder particles adhere to the surface, resulting in a poor surface finish. Some non-melted powder particles may be trapped in the deposited layers during the process, thus reducing the mechanical property [28].

**Figure 4:**
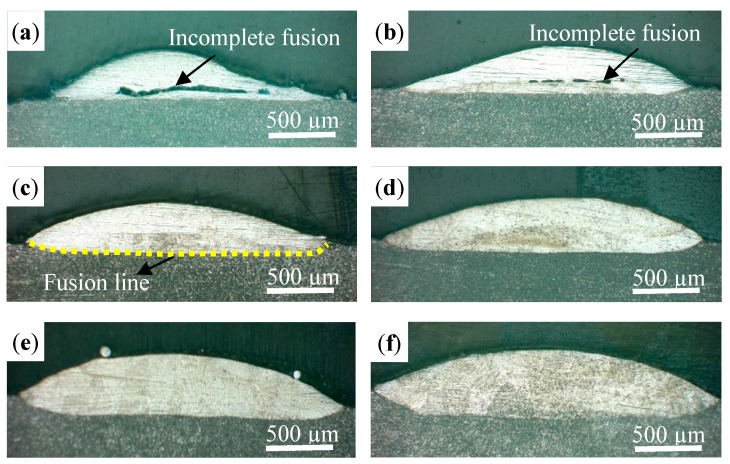
The morphologies of the cross-section with different laser powers: (**a**) 700 W, (**b**) 800 W, (**c**) 900 W, (**d**) 1000 W, (**e**) 1100 W, and (**f**) 1200 W.

**Figure 5:**
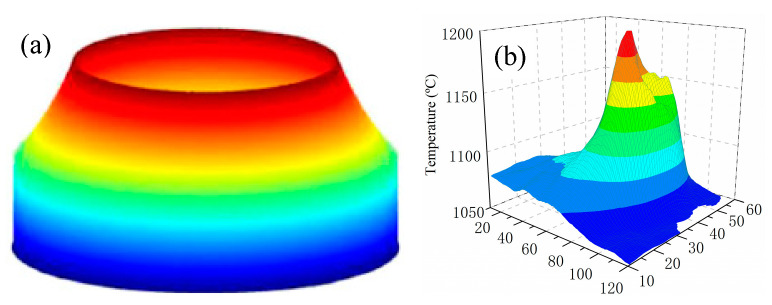
The energy intensity and temperature distribution: (**a**) energy intensity of the annular laser beam [29]; (**b**) 3D temperature distribution of the molten pool [20].

The defects in the track, such as pores, microcracks, incomplete fusion, and cavities, were detrimental to the resultant properties. Figure 4 shows the OM images of the cross-sections of the tracks at different laser powers. The laser power had the greatest influence on the morphology. As the laser power was less than 800 W, the track was asymmetric, and the large-sized incomplete fusion defect can be observed in Figure 4a,b. The incomplete fusion defects were primarily distributed in the bottom of the track. This is mainly because the energy intensity of the annular laser presents no energy at the center, while a it has a bimodal profile on the outside (Figure 5a) [29], and the central energy needs to be transferred through the heat conduction and heat convection of the external energy. Figure 5b shows the temperature distribution of the molten pool measured by an infrared imaging device; the distribution indicates that the temperature at the edge is a little bit higher than in the middle [20]. When the laser power was low, the convection in the molten pool and the powder melting were insufficient, and thus the incomplete fusion defects occurred. It can also be observed in Figure 4a that the track was not metallurgically bonded to the substrate. In Figure 4c–f, it can be seen that the tracks are symmetric and have no incomplete fusion defects as the laser power increases. In particular, the fusion line generated by the annular laser is straight (yellow dotted line) and is different from the “U” shaped fusion line produced by a solid Gaussian laser. This is mainly related to the uniform temperature distribution of the molten pool; that is, the temperature is effectively increased at the boundary and slightly weakened at its center, as shown in Figure 5b.

**Figure 6:**
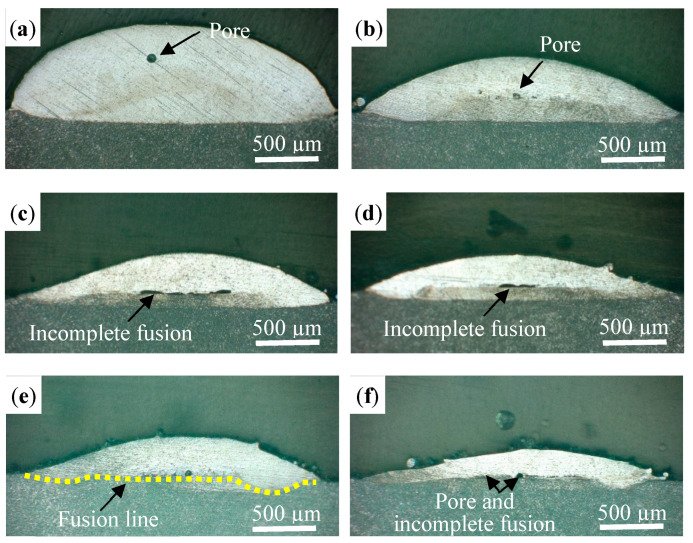
The morphologies of the cross-section with different scanning speeds: (**a**) 3 mm/s, (**b**) 4 mm/s, (**c**) 5 mm/s, (**d**) 6 mm/s, (**e**) 7 mm/s, and (**f**) 8 mm/s.

Figure 6 shows the morphologies of the cross-section of the tracks at different scanning speeds. The pores and incomplete fusion defects can be observed in the tracks. When the scanning speed is in the range of 3~6 mm/s, the tracks have a good symmetry, and the fusion lines are relatively straight. However, as the scanning speed exceeds 7 mm/s, the tracks begin to become asymmetrical, and the fusion line presents a “U” shape on both sides and no fusion in the middle. The shape of the fusion line corresponds with the analysis of the energy intensity of the annular laser. High scanning speed shortened the lifetime of the molten pool, and weakened the convection of the molten pool, resulting in a poor forming quality. A large-sized incomplete fusion defect (as shown in Figure 6c–f) in the track was detrimental to a good bonding between the layers during the layer-by-layer forming process, which caused the stress concentration there to greatly reduce the performance of the formed parts [30].

**Figure 7:**
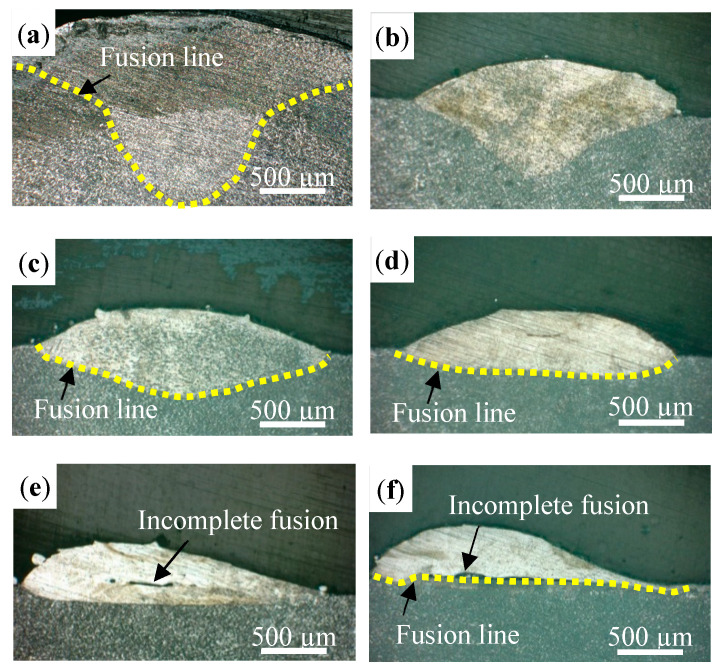
The morphologies of the cross-section with different defocus distances: (**a**) 0 mm, (**b**) −1 mm, (**c**) −2 mm, (**d**) −3 mm, (**e**) −4 mm, and (**f**) −5 mm.

The morphologies of the cross-section of the tracks at different defocus distances are shown in Figure 7. It can be observed that the defocus distance has a great influence on the morphology of the track. The defocus distance varies from 0 mm to −5 mm; the shape of the fusion line changes from a “V” shape (Figure 7a,b) to a “U” shape (Figure 7c), then to a straight line (Figure 7d,e), and finally to a “U” shape on both sides, with no fusion in the middle (Figure 7f). This is mainly related to the laser energy distribution at different defocus distances. For a defocus distance of 0 mm (that is, the focal position), the laser spot is solid with a Gaussian energy distribution, which has a high energy in the center and a low energy on both sides, eventually leading to a V-shaped fusion line. With the further increase in the defocus distance (−2 to −4 mm), the laser spot becomes an annular shape with a double Gaussian on both sides and no energy at the center. In the deposition process, the temperature distribution is relatively uniform; so, the fusion line changes from a “U” shape to a straight line. When the defocus distance reached −5 mm, the laser energy density was low due to a large laser spot, and a lack of bonding between the track and the substrate occurred at the center.

### 3.2. Effect of Process Parameters on Geometrical Characteristics

**Figure 8:**
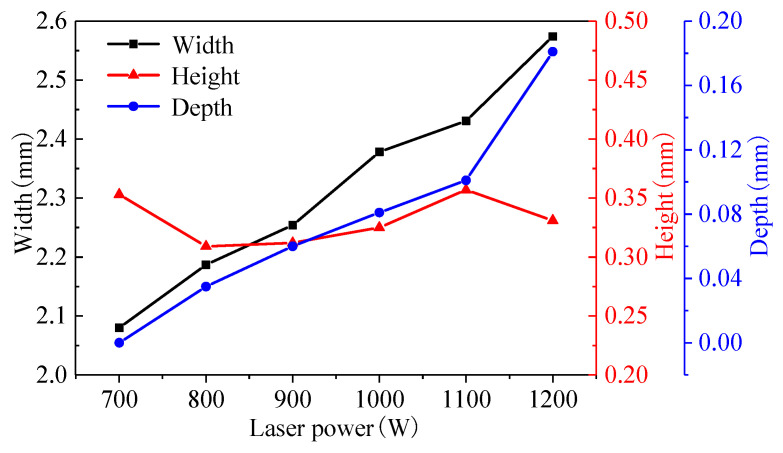
Track geometry at different laser powers.

The bead width, bead height, and fusion depth were used to analyze the geometrical characteristics of the track, which obviously affected the metallurgical bonding of the adjacent tracks or layers. Figure 8 shows the track geometry under different laser powers. It can be seen that as the laser power increases from 700 W to 1200 W, the bead width increases from 2.08 mm to 2.574 mm, the bead height fluctuates between 300 and 360 µm, and the fusion depth increases from 0 µm to 181 µm. The bead width and bead height have an approximately linear relationship with laser power.

**Figure 9:**
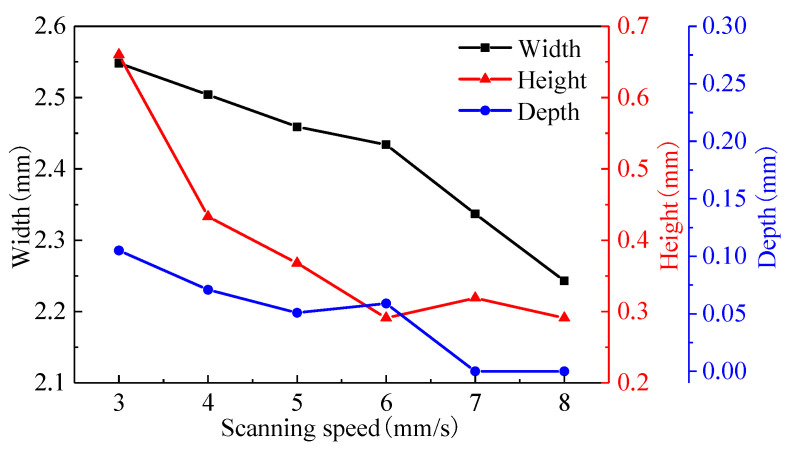
Track geometry at different scanning speeds.

The width, height, and fusion depth of the single track at different scanning speeds are shown in Figure 9. When the scanning speed increases from 3 mm/s to 6 mm/s, the bead width decreases from 2.548 mm to 2.434 mm, the bead height decreases from 660 µm to 291 µm, and the fusion depth fluctuates between 50 and 110 µm. When the scanning speed increases from 6 mm/s to 8 mm/s, the speed decreasing rate accelerates and the bead width decreases from 2.434 mm to 2.183 mm, while the bead height hardly changes, staying between 240 µm and 320 µm. The fusion depth becomes zero, and there is no metallurgical bonding between the track and the substrate due to low heat input.

**Figure 10:**
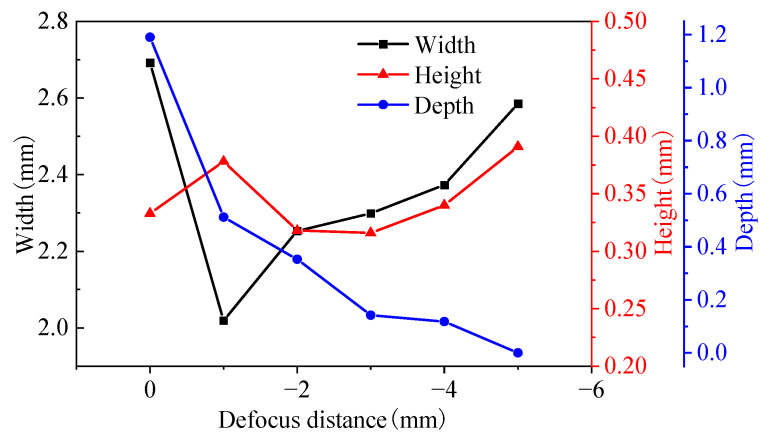
Track geometry at different defocus distances.

Figure 10 shows the bead width, height, and depth at different defocus distances. The defocus distance had the greatest influence on the geometric characteristics. When the defocus distance increased from −1 mm to −4 mm, the bead width increased from 2.019 mm to 2.373 mm due to the increasing of the laser spot. The fusion depth decreased from 512 µm to 118 µm, and the bead height fluctuated between 300 and 400 µm. For the focus position, the bead width reached 2.692 mm, and the fusion depth was 1190 µm, which was higher than that of the negative defocus distance. This may be related to the oxygen content during the ALMD process. At the focus point, the laser energy is higher, and the inert atmosphere generated by the double-layer shielding device cannot effectively protect the molten pool. The oxygen in the local atmosphere can reduce the surface tension of the molten pool and thus spread the molten pool and increase the width of the molten pool. In addition, the peak energy of the center of the laser spot is high, and thus the fusion depth is high. When the defocus distance reached −5 mm, there was no metallurgical bonding and the fusion depth was 0.

### 3.3. Effect of Process Parameters on Thermal History

**Figure 11:**
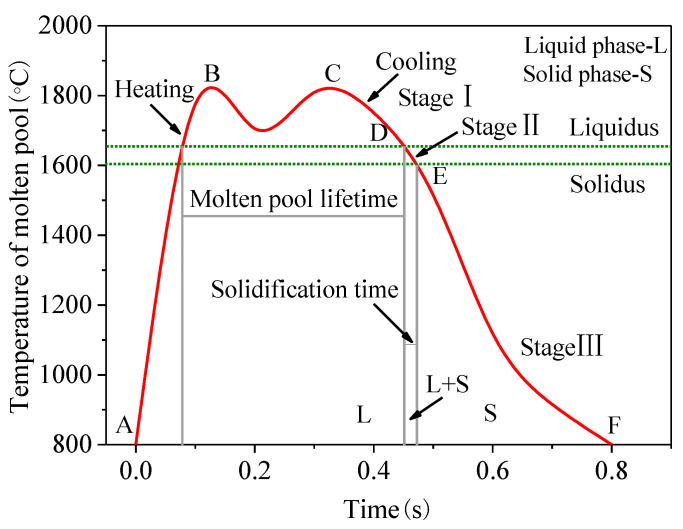
Typical temperature variation curve of molten pool.

**Figure 12:**
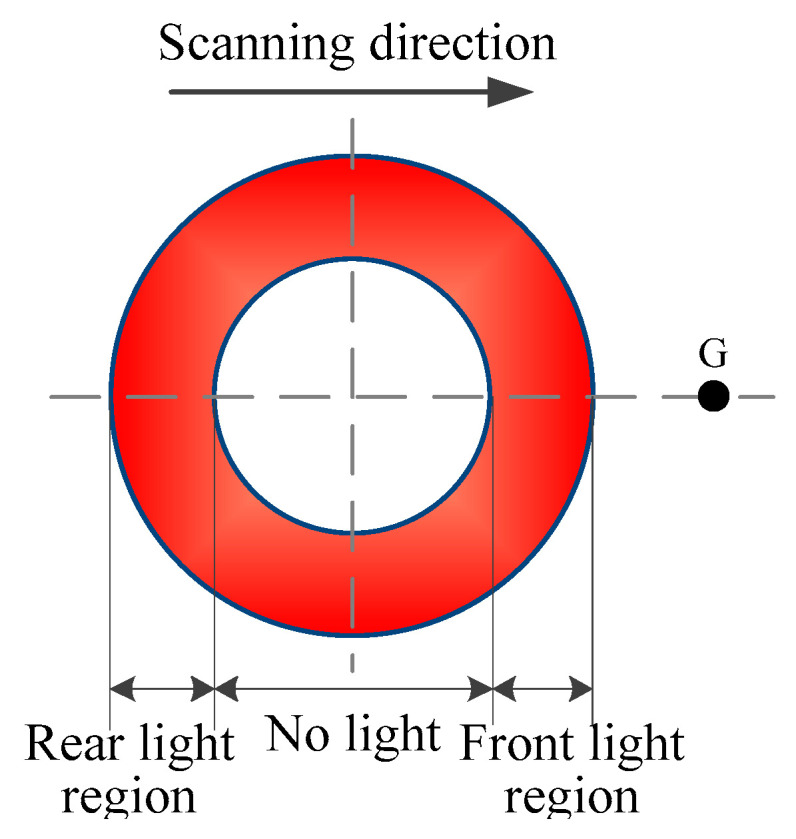
Schematic diagram of thermal loading of annular laser beam at fixed point G.

The thermal history of the molten pool during the ALMD process is related to various process parameters which affect the formability. Figure 11 shows the typical temperature variation curve of the molten pool produced by ALMD at a fixed point. It can be seen that the molten pool undergoes two heating and cooling processes, which are different from the traditional heating and cooling processes generated by Gaussian thermal loading [31]. During the heating stage (AC line), the molten pool temperature rises rapidly. There are two peak temperatures (point B and point C) for the molten pool. This is mainly due to a bimodal energy distribution of the annular beam where it has no light in the middle zone (white area) and has light in the exterior zone (red area), as shown in Figure 12. Along the scanning direction, fixed point G first experiences the radiation in the front light region of the annular laser, and the temperature rises to the first peak temperature at point B. Then, point G enters the dark region, and the temperature begins to drop due to no laser radiation. However, the temperature drop is small due to the effect of heat conduction. Then, point G enters the rear light region of the annular laser, and the temperature rises again to reach the second peak temperature at point C. Therefore, in the moving process of the annular laser beam, fixed point G undergoes two occurrences of laser thermal loading, which results in a temperature process of rise–drop–rise–drop.

The cooling stage (CF line) consists of three parts, namely the CD stage, the DE stage, and the EF stage. The first cooling stage (CD) is liquid phase cooling, in which the temperature is above the liquidus (1655 °C) and the molten pool is completely liquid. The second cooling stage (DE) is between the liquidus and solidus. The molten pool solidifies from point D and ends at point E. At this stage, the liquid phase and solid phase coexist, and the cooling rate determines the microstructure characteristics, which can be used to analyze and regulate the microstructure [32]. The third cooling stage (EF) is solid phase cooling, where the temperature is below the solid phase line (1605 °C) and the molten pool is completely solid. According to the molten pool temperature curve, the molten pool parameters, such as the molten pool lifetime, solidification time, and cooling rate, are analyzed.

**Figure 13:**
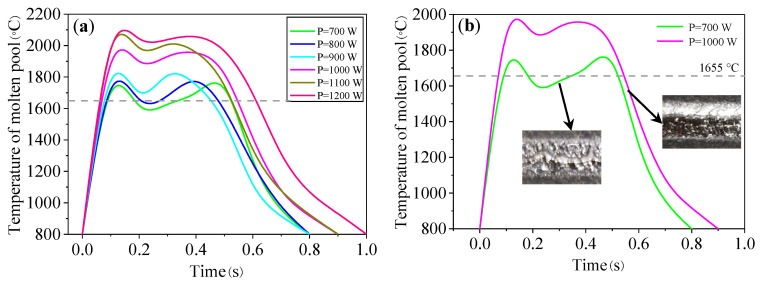
(**a**) Temperature curve of molten pool under different laser powers; (**b**) temperature curve at low and high laser power.

Figure 13 shows the temperature curves of the molten pool at the different laser powers given in Table 2. As can be seen from Figure 13a, the laser power has a significant impact on the molten pool temperature. With the increase in the laser power, the peak temperature of the molten pool increases. When the laser power is low (700~800 W), the peak temperature is low. When the annular laser beam entered the dark region, the temperature was lower than that of the liquid phase, and thus the defective tracks were formed due to insufficient molten pool convection and powder melting, as shown in the green curve of Figure 13b. When the laser power increased, the temperature of the molten pool was always higher than that of the liquidus during the whole process. The obtained tracks were smooth and no obvious macroscopic defects were observed, as shown in the pink curve of Figure 13b.

**Figure 14:**
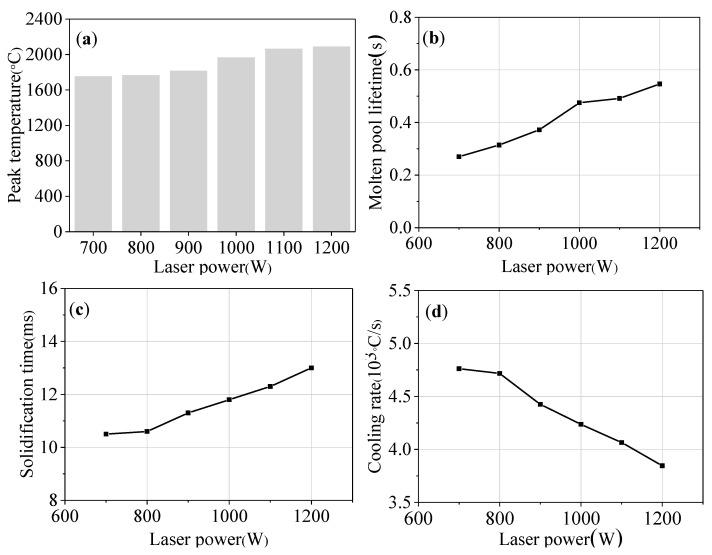
The parameters of molten pool under different laser powers: (**a**) peak temperature; (**b**) molten pool lifetime; (**c**) solidification time; and (**d**) cooling rate.

The parameters of the molten pool at different laser powers are shown in Figure 14. When the laser power increased from 700 W to 1200 W, the peak temperature of the molten pool increased from 1761 °C to 2097 °C (as shown in Figure 14a). This is mainly because the increase in laser power improved the heat input of the laser energy. In addition, the molten pool lifetime increased from 0.27 s to 0.55 s (as shown in Figure 14b), the solidification time increased from 10.5 ms to 13 ms (as shown in Figure 14c), and the cooling rate decreased from 4.76 × 10^3^ °C/s to 3.85 × 10^3^ °C/s (as shown in Figure 14d).

**Figure 15:**
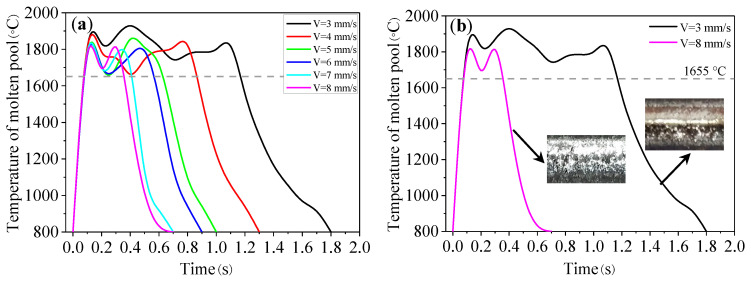
(**a**) Temperature curve of molten pool under different scanning speeds; (**b**) temperature curve at low and high scanning speeds.

The temperature curves of the molten pool at different scanning speeds are shown in Figure 15. It can be seen from Figure 15a that, compared with laser power, scanning speed has a significant influence on the shape of the temperature curve. The scanning speed has the greatest influence on the thermal history. As the scanning speed increases, the temperature curve becomes steeper and the thermal cycle becomes shorter. At a low scanning speed (3~6 mm/s), the peak temperature will be stable for a period of time, which is conducive to the convection of the molten pool and powder melting, as shown in the black curve of Figure 15b. However, if the scanning speed is too small, the high temperature zone will exceed the inert gas protection range, resulting in the oxidation of the track. As can be seen from the illustration in Figure 15b, the surface of the track appears as a light yellow color, and a small degree of oxidation occurs at the scanning speed of 3 mm/s. At the high scanning speeds (7~8 mm/s), the molten pool temperature rises and falls rapidly, as shown in the pink curve of Figure 15b.

**Figure 16:**
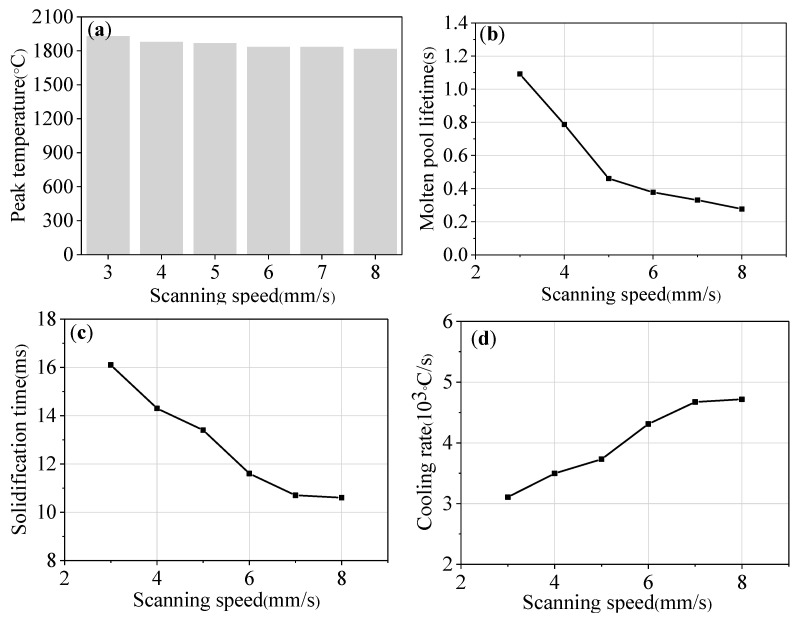
The parameters of molten pool under different scanning speeds: (**a**) peak temperature; (**b**) molten pool lifetime; (**c**) solidification time; and (**d**) cooling rate.

Figure 16 shows the parameters of the molten pool at different scanning speeds. It can be seen from Figure 16a that the scanning speed has little influence on the peak temperature of the molten pool. Although the input energy per unit time increases as the scanning speed decreases, the amount of powder delivery also increases. The powder will absorb partial energy; so, the peak temperature fluctuates less. As can be seen from Figure 16b–d, as the scanning speed increases from 3 mm/s to 8 mm/s, the molten pool lifetime decreases from 1.09 s to 0.28 s, the solidification time decreases from 16.1 ms to 10.6 ms, and the cooling rate increases from 3.11 × 10^3^ °C/s to 4.72 × 10^3^ °C/s. A higher scanning speed can result in a lower molten pool lifetime and solidification time as well as a higher cooling rate.

**Figure 17:**
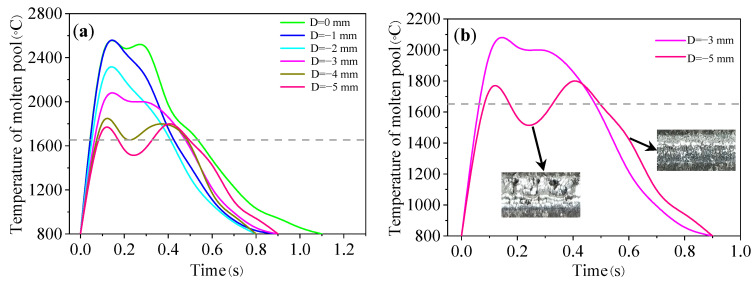
(**a**) Temperature curve of molten pool under different defocus distances; (**b**) temperature curve at low and high defocus distances.

Figure 17 shows the temperature curves of the molten pool at different defocus distances. It can be seen from Figure 17a that the defocus distance has a significant impact on the molten pool temperature. With the increase in defocus distance, the peak temperature decreases. When the defocus distance was −5 mm, a uniform and continuous track could not be formed due to low laser energy density, as shown in the red curve of Figure 17b.

**Figure 18:**
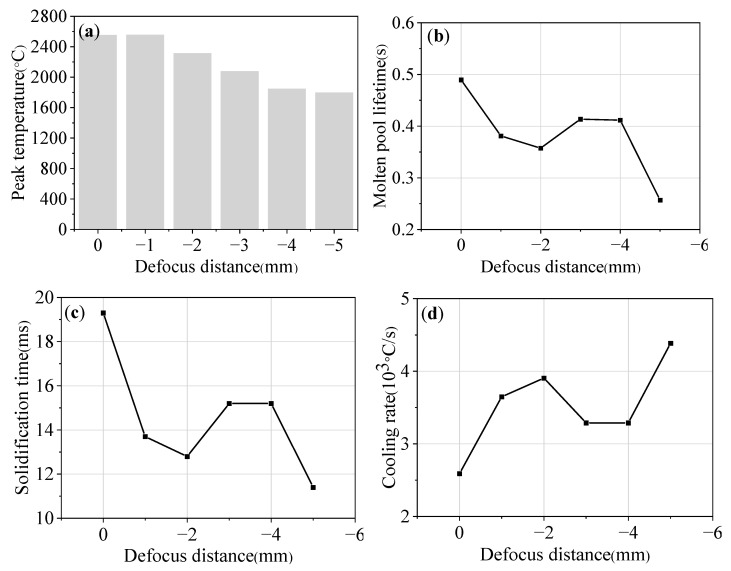
The parameters of molten pool under different defocus distances: (**a**) peak temperature; (**b**) molten pool lifetime; (**c**) solidification time; and (**d**) cooling rate.

Figure 18 shows the parameters of the molten pool at different defocus distances. When the defocus distance is 0~−1 mm, that is, near the focal point, the peak temperature is high (about 2560 °C). When the defocus distance increases from −2 mm to −5 mm, the peak temperature decreases from 2316.6 °C to 1800 °C. When the defocus distance increases from 0 mm to −5 mm, the molten pool lifetime decreases from 0.49 s to 0.26 s, the solidification time decreases from 19.3 ms to 11.4 ms, and the cooling rate increases from 2.59 × 10^3^ °C/s to 4.39 × 10^3^ °C/s.

**Figure 19:**
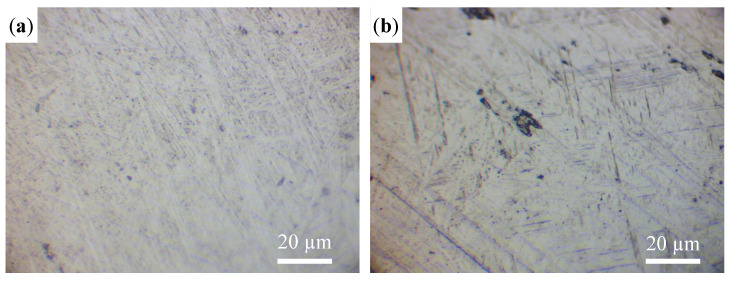
The microstructure under different defocus distances: (**a**) 0 mm; (**b**) −4 mm.

In order to compare the non-annular and annular LMD, the microstructures of the tracks under defocus distances of 0 mm and −4 mm, representing the non-annular and annular laser, respectively, were observed and are shown in Figure 19. The microstructure is an elongated martensitic α’ phase, but the α´ martensite produced by the annular LMD is much finer. This is mainly related to the cooling rate [18]. It can be seen from Figure 17 and Figure 18 that the peak temperature of the non-annular LMD is higher than that of the annular LMD and that the cooling rate is lower.

### 3.4. Single Wall Characteristics

**Figure 20:**
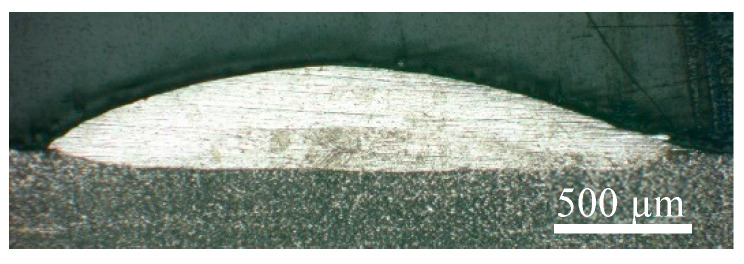
The morphology of the cross-section of the track with optimal process parameters.

**Figure 21:**
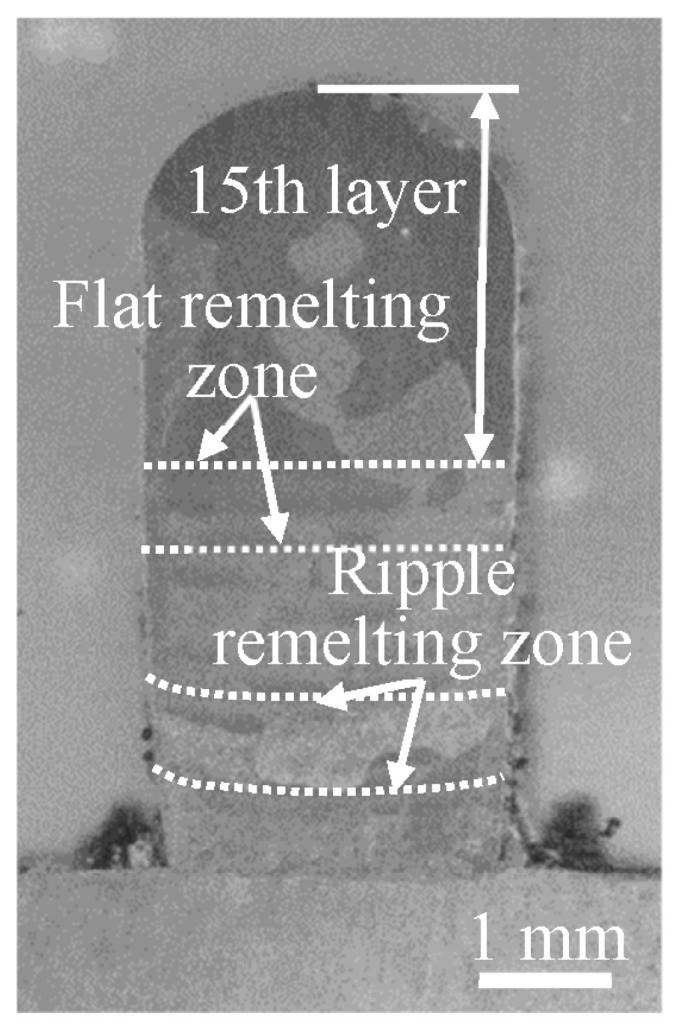
The macrostructure of cross-section of the thin wall sample.

The optimal process parameters of the single track were as follows: the laser power was 1050 W, the scanning speed was 5 mm/s, the defocus distance was −3.5 mm, the powder feeding rate was 1.4 g/min, and the total shielding gas flow rate was 30 L/min. The resultant morphology of the cross-section of the track is shown in Figure 20. In the ALMD process, the laser processing of the current layer had a thermal effect on the deposited layers. A single 15-layer thin wall sample was produced using the above optimal process parameters. The macrostructure of the cross-section of the sample is shown in Figure 21. The prior β grain can be observed. The remelting band can also be seen, and it changes from a ripple at the bottom to flat at the top, indicating that the thickness of the deposited layer tends to stabilize with the increase in deposited layers (about 400 µm thickness per layer). Furthermore, the melting depth of the 15th layer is 2.85 mm, indicating that approximately seven of the deposited layers were remelted under this process.

**Figure 22:**
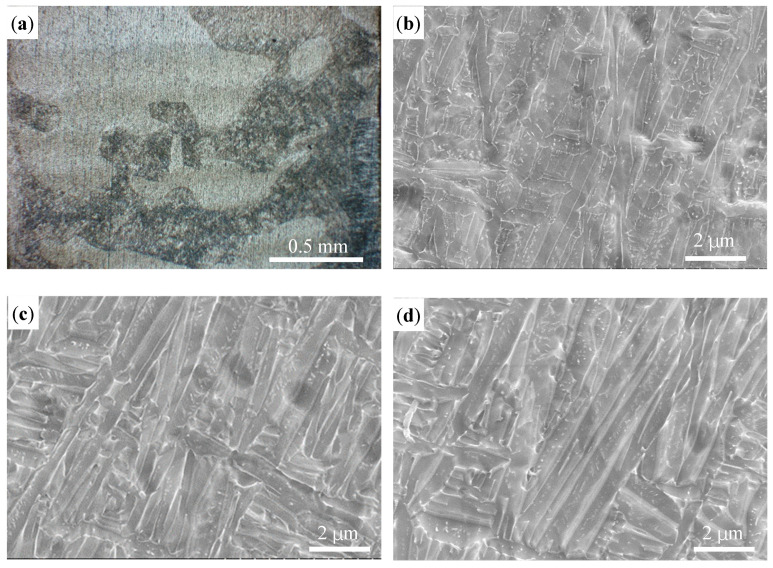
The microstructure of the thin wall sample: (**a**) OM image in the middle; (**b**) SEM image on the top; (**c**) SEM image in the middle; and (**d**) SEM image on the bottom.

**Figure 23:**
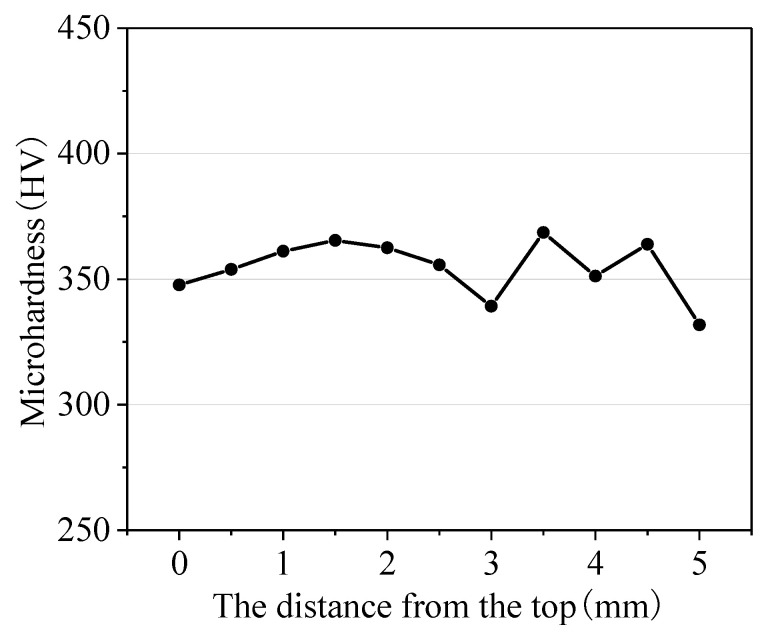
Microstructure of the thin wall sample.

Figure 22 shows the microstructure of the thin wall sample. The OM image of the central position of the sample is shown in Figure 22a. The layers appear as alternate dark and bright phases; this alternation is mainly related to crystal grain orientation [33]. It can be observed from Figure 22b–d that the microstructures at the different positions are similar and consist of a fine lath α phase and β phase. Many α clusters are distributed within the crystal. However the size varies at the different positions, and the clusters on the top position are of a short length and small width. The microhardness of the sample from the top to the bottom is shown in Figure 23. The microhardness fluctuates slightly, ranging from 330 HV to 370 HV. 

## 4. Conclusions

This study experimentally investigated the influence of laser power, scanning speed, and defocus distance on the geometry and thermal history of the Ti6Al4V alloy by the ALMD process. The microstructure and microhardness of the thin wall sample were also examined. The following conclusions can be drawn:

1. For low laser power, high scanning speed, or defocus distance, discontinuous and uneven tracks were observed, as well as pores and large-sized incomplete fusion defects in the track. A slight oxidation on the surface of the track could be observed when the input energy was too high.

2. The fusion line produced by the annular laser was straight with the appropriate process parameters; thus, it differed from the “U” shape generated by the solid Gaussian laser; this was due to the uniform temperature distribution of the molten pool. The defocus distance had a great influence on the shape of the fusion line, changing from a “V” shape to a “U” shape, then to a straight line when the defocus distance varied from 0 mm to −4 mm.

3. The bead width and height increased linearly with the laser power. The scanning speed had a significant effect on the bead height, and the fusion depth was sensitive to the defocus distance.

4. The laser power had a significant impact on the molten pool temperature, and the scanning speed obviously affected the shape of the temperature curve. When the laser power was less than 800 W or the defocus distance was greater than −5 mm, the temperature of the molten pool was lower than the liquid phase temperature of the Ti6Al4V material, which led to the occurrence of defective tracks. Compared to the laser power and defocus distance, the scanning speed was the most affecting parameter for the molten pool lifetime and the solidification time, as well as the cooling rate.

5. The optimal process parameters were a laser power of 1050 W, a scanning speed of 5 mm/s, a defocus distance of −3.5 mm, a powder feeding rate of 1.4 g/min, and a total shielding gas flow rate of 30 L/min. The microstructures of different zones were similar and consisted of a fine lath α phase and β phase, but the size varied, and the α clusters on the top position were of short length and small width. The microhardness ranged from 330 HV to 370 HV.

Future research will control the temperature of the interlayered molten pool during the deposition process by adjusting the process parameters.

## Figures and Tables

**Table 1:** Chemical compositions of used materials (wt%).

Material	Al	V	Fe	O	C	N	H	Ti
Substrate	6.20	4.30	0.19	0.1800	0.02	0.0100	0.0020	Bal.
Powder	6.36	4.06	0.05	0.0785	0.01	0.0028	0.0012	Bal.

**Table 2:** Process parameters for ALMD experiments.

Exp. No.	Laser Power (W)	Scanning Speed (mm/s)	Defocus Distance (mm)
1	700~1200	5	−3.5
2	1000	3~8	−3.5
3	1000	5	0~−5

## Data Availability

Data sharing is not applicable.
